# Association between brain serotonin 4 receptor binding and reactivity to emotional faces in depressed and healthy individuals

**DOI:** 10.1038/s41398-023-02440-3

**Published:** 2023-05-11

**Authors:** Anjali Sankar, Brice Ozenne, Vibeke H. Dam, Claus Svarer, Martin B. Jørgensen, Kamilla W. Miskowiak, Vibe G. Frokjaer, Gitte M. Knudsen, Patrick M. Fisher

**Affiliations:** 1 Neurobiology Research Unit, Copenhagen University Hospital Rigshospitalet, Copenhagen, Denmark; 2 Department of Public Health, Section of Biostatistics, University of Copenhagen, Copenhagen, Denmark; 3 Department of Clinical Medicine, University of Copenhagen, Copenhagen, Denmark; 4 Psychiatric Center Copenhagen, Copenhagen University Hospital Rigshospitalet, Copenhagen, Denmark; 5 Neurocognition and Emotion in Affective Disorders (NEAD) Centre, Mental Health Services, Capital Region of Denmark, and Department of Psychology, University of Copenhagen, Copenhagen, Denmark; 6 Department of Psychology, University of Copenhagen, Copenhagen, Denmark; 7 Department of Drug Design and Pharmacology, University of Copenhagen, Copenhagen, Denmark

**Keywords:** Molecular neuroscience, Depression

## Abstract

Brain serotonergic (5-HT) signaling is posited to modulate neural responses to emotional stimuli. Dysfunction in 5-HT signaling is implicated in major depressive disorder (MDD), a disorder associated with significant disturbances in emotion processing. In MDD, recent evidence points to altered 5-HT_4_ receptor (5-HT_4_R) levels, a promising target for antidepressant treatment. However, how these alterations influence neural processing of emotions in MDD remains poorly understood. This is the first study to examine the association between 5-HT_4_R binding and neural responses to emotions in patients with MDD and healthy controls. The study included one hundred and thirty-eight participants, comprising 88 outpatients with MDD from the NeuroPharm clinical trial (ClinicalTrials.gov identifier: NCT02869035) and 50 healthy controls. Participants underwent an [^11^C]SB207145 positron emission tomography (PET) scan to quantify 5-HT_4_R binding (BP_ND_) and a functional magnetic resonance imaging (fMRI) scan during which they performed an emotional face matching task. We examined the association between regional 5-HT_4_R binding and corticolimbic responses to emotional faces using a linear latent variable model, including whether this association was moderated by depression status. We observed a positive correlation between 5-HT_4_R BP_ND_ and the corticolimbic response to emotional faces across participants (*r* = 0.20, *p* = 0.03). This association did not differ between groups (parameter estimate difference = 0.002, 95% CI = −0.008: 0.013, *p* = 0.72). Thus, in the largest PET/fMRI study of associations between serotonergic signaling and brain function, we found a positive association between 5-HT_4_R binding and neural responses to emotions that appear unaltered in MDD. Future clinical trials with novel pharmacological agents targeting 5-HT_4_R are needed to confirm whether they ameliorate emotion processing biases in MDD.

## Introduction

The brain serotonin (5-HT) system plays a crucial role in processing emotionally salient stimuli [1, 2]. Brain 5-HT signaling appears to be altered in major depressive disorder (MDD), a disorder that is associated with significant abnormalities in processing emotionally salient stimuli. Prior positron emission tomography (PET) studies have shown alterations in multiple serotonin receptors (e.g., 5-HT_1A,_ and 5-HT_2A_ receptors), and in the serotonin transporter (5-HTT) in MDD [3–6]. However, the direction of alterations reported in these studies have been mixed. For instance, studies have reported both increased [3, 4, 7, 8] as well as decreased [5, 9–13] binding to the 5-HT_1A,_ and 5-HT_2A_ receptors, and to the 5-HTT, in MDD. The differing results may largely reflect methodological inconsistencies such as different outcome measures [14], but may also reflect differing clinical characteristics of the sample, such as medication and remission status [13]. More recently, evidence from the largest clinical PET trial investigating the brain serotonin 4 receptor (5-HT_4_R) showed decreased binding in patients with MDD relative to healthy controls [15]. Although no drugs targeting 5-HT_4_R are approved to treat MDD, the study of the 5-HT_4_R system is of particular interest as preclinical studies point to the 5-HT_4_R as being a promising novel target for fast-acting antidepressant treatment [16, 17].

Underlying disturbances in processing emotionally salient stimuli are dysfunctions in cortico-limbic brain regions subserving emotion identification and regulation processes such as the amygdala, insula, ventromedial prefrontal cortex (vmPFC), and the dorsolateral prefrontal cortex (dlPFC). Meta-analyses of functional magnetic resonance imaging (fMRI) employing emotion processing tasks have consistently shown activation of the amygdala and insula, regions that are important for emotion identification, during processing of emotional stimuli [18–21]. fMRI studies have also shown recruitment of the vmPFC and the dlPFC, regions which are important for regulating responses during processing of emotional stimuli [20, 22, 23]. In patients with MDD, dysfunctional responses to emotional stimuli in the amygdala, insula, vmPFC, and dlPFC have been observed relative to healthy controls [24–27], however factors such as emotional valence [24], type of stimuli (i.e., whether facial or non-facial affective stimuli) [21], cognitive load [28], and task instruction (i.e., whether the emotion is implicitly or explicitly processed) [21] are known to significantly influence the pattern of activations.

Multimodal brain imaging integrating PET and fMRI provides a powerful framework for establishing associations between specific neurotransmitter signaling pathways and distributed functional neural activity [29]. Previous such studies have shown that brain serotonergic signaling (for instance, via 5HT_1A_ and 5HT_2A_ receptors) modulate neural responses to emotional stimuli [30–34]. In healthy individuals, changes in brain 5-HT_4_R binding in response to a 3-week intervention with SSRI are associated with corresponding changes in amygdala reactivity [35]. It remains to be investigated if the coupling between serotonergic signaling via the 5-HT_4_R and emotion-processing related neural responses is present at baseline, and if this relationship is preserved in MDD. A better understanding of the association between 5-HT_4_R binding and neural responses to emotionally salient stimuli in patients with MDD could shed light on the pathophysiology underlying the disorder. Such evidence could also support the use of new pharmacological treatments that work on the 5-HT_4_R which could help modulate the neural responses underlying aberrant emotional processing in MDD. Targeting aberrant emotional processing in patients with MDD is important because it is a risk factor for subsequent depressive episodes and suicidality [36, 37], as well as for recurrence and relapse in remitted individuals [38]. With this objective, the goal of this study is to utilize PET in combination with task-based fMRI to investigate the association between 5-HT_4_R binding and functional responses in brain regions subserving emotion processing in individuals, and further investigate whether this association is moderated by diagnostic status. We hypothesized that patients with MDD would show a decoupling between 5-HT_4_R binding and neural responses to emotionally salient cues relative to healthy individuals, which might play a role in the pathophysiology of the disorder.

## Subjects and methods

### Participants

The patients with MDD included in this study were from the NeuroPharm cohort, an open-label clinical depression trial [39]. Eighty-eight of these patients completed both an [^11^C]SB207145 PET and MRI (including the emotional face fMRI paradigm) scan and were included in this study. All patients met DSM-5 and ICD-10 criteria for MDD according to the Mini International Neuropsychiatric Interview (M.I.N.I) [40] and were recruited from the mental health services in the capital region of Denmark. All diagnoses were confirmed by a specialist in Psychiatry. Patients additionally met criteria for moderate-severe depression, as defined by a score of at least 17 on the 17-item Hamilton Depression Rating Scale (HAMD_17_) [41] and were free of antidepressant medication for at least two months before entry into the study. Exclusion criteria for patients with MDD included: diagnosis with primary axis I psychiatric disorders other than MDD, current depressive episode exceeding two years, more than one prior antidepressant treatment during current depressive episode, previous non-responsiveness or known contraindications to SSRIs, acute suicidal ideation or psychosis, severe somatic illness, pregnancy, breast-feeding, use of psychotropic drugs affecting the central nervous system that cannot be washed out prior to study entry, history of severe brain injury, severe intellectual impairments, contraindications to PET or MR, exposure to radioactivity >10 mSv within the last year, and insufficient knowledge of the Danish language to complete procedures of the study [39]. Severity of patients’ depression was assessed using the clinician rated HAMD_17_ and the self-reported Major Depression Inventory (MDI) [42, 43].

The NeuroPharm clinical trial (NCT02869035) was approved by the local scientific ethics committee (H-15017713), the Danish Data Protection Agency, and Danish Medicines Agency (NeuroPharm-NP1, EudraCT number: 2016-001626-34). The study was conducted in accordance with the Declaration of Helsinki II, and Good Clinical Practice (GCP) guidelines.

Fifty-two healthy controls with no axis I psychiatric disorder were included for comparisons. They were either recruited from an online recruitment site, or drawn from the Cimbi database, a quality-controlled central repository of the Neurobiology Research Unit, Rigshospitalet, Copenhagen [44]. The healthy controls underwent the same PET and MRI protocols (i.e. [^11^C]SB207145 PET and emotional faces fMRI paradigm) on the same scanners as the patients with MDD. The exclusion criteria for healthy controls were the same as patients, and additionally they were without a lifetime history of both Axis-I psychiatric illness and psychotropic medications [39].

All participants provided written informed consent prior to participation.

### Genotyping

Genotyping of the short (S) and long (L) 5-HTTLPR variants was performed on all participants using methods described previously [45, 46]. For the analysis performed herein, participants were classified as either L_A_L_A_ homozygous or S′ carriers.

### MRI data acquisition

High resolution 3D T1-weighted Magnetization-Prepared Rapid Acquisition Gradient Echo (MPRAGE) structural scans were acquired at Rigshospitalet (Copenhagen, DK) on a Siemens Magnetom 3T Prisma scanner (Erlangen, DE) using a 64-channel head/neck coil with the following parameters; repetition time = 1900 ms, echo time= 2.58 ms, inversion time = 900 ms, flip angle = 9°, in-plane matrix = 256 × 256, in-plane Resolution= 0.9 × 0.9 mm, 224 slices and a slice thickness of 0.9 mm, no gap. Blood-oxygen level dependent (BOLD) fMRI scans were acquired while performing the emotional faces paradigm using a T2*-weighted gradient echo-planar imaging (EPI) sequence with the following parameters: repetition time = 2000 ms, echo time = 30 ms, flip angle = 90°, in-plane matrix = 64 × 64 mm, in-plane resolution = 3.6 × 3.6 mm, 32 slices and a slice thickness = 3.0 mm, gap = 0.75 mm. A corresponding gradient-echo field map was acquired to correct for spatial distortions (repetition time = 400 ms, 7.38 ms).

### Emotional faces paradigm

The BOLD fMRI paradigm used herein has been described previously [47, 48]. Briefly, this version of the emotional faces paradigm consisted of four experimental blocks of emotional faces stimuli (fear, anger, neutral, and surprise) interleaved with five control blocks of geometric shapes (circles, and ellipses). Prior to each block, a brief instruction statement “Match Shapes” or “Match Faces”, lasting two seconds was presented. Each block comprised of six trials wherein they viewed a trio of either faces or shapes. With a button press, participants were asked to respond which one of two shapes/faces at the bottom of the screen matched the target shape/face at the top of the screen as quickly and accurately as they could. Each experimental block (i.e., faces block) expressed only one of the four emotion types and were ordered randomly across participants. Faces stimuli were presented for four seconds with a variable inter-stimulus interval (two, four or six seconds, mean duration= four seconds). Shapes were also presented for four seconds and had a fixed inter-stimulus interval of two seconds. The paradigm was presented and behavioral responses were recorded using E-prime (Psychological Software Tools, Pittsburgh, PA, USA).

As done in previous studies [48], patients with MDD and healthy controls were removed from further analyses if they performed with less than 80% accuracy on the task. Two healthy controls did not meet the aforementioned inclusion criterion and were excluded for the final analyses.

### MRI processing and analyses

Functional MRI data were preprocessed and analyzed using the Statistical Parametric Mapping 12 software (SPM12, https://www.fil.ion.ucl.ac.uk/spm/software/spm12/). Functional images were corrected for slice-timing, spatially realigned, corrected for spatial distortions and co-registered to the individual’s T1-weighted MPRAGE image. The Automated Anatomical Labeling (AAL3) atlas [49] in Montreal Neurologic Institute (MNI) standard space was warped to each individual participants’ native space using the inverse deformation field generated during segmentation of the T1-weighted MPRAGE image. Functional images were smoothed with a 4 mm full-width half-maximum Gaussian filter. Artefact Detection Toolbox (ART) http://www.nitrc.org/projects/artifact_detect) was used to identify and censor individual volumes with excessive motion (>2 mm) or variation (>4 SDs).

The General Linear Model (GLM) [50] was applied to the time series. A high pass filter of 128 s was applied to control for slow-frequency fluctuations and improve signal-to-noise. The stimuli presentation period was modeled as a boxcar convolved with the canonical hemodynamic response function (HRF), resulting in five regressors pertaining to fear, anger, neutral, surprises faces, and shapes stimuli. The GLM included the six motion parameters and censored volumes as nuisance regressors. The primary contrast of interest for this study was all faces versus all shapes, and was used for all analyses reported.

### PET data acquisition

The PET data acquisition procedures are described in detail elsewhere [35, 48, 51]. Briefly, scans were conducted using a high-resolution research tomography (HRRT) Siemens scanner (256 × 256 × 207 voxels; 1.22 × 1.22 × 1.22 mm). All participants underwent a 6 min transmission scan and received an intravenous bolus injection of ~600 MBq of [^11^C]SB207145. The bolus was administered over 20 s followed by 120 min acquisition of dynamic PET data.

### PET processing and quantification

Motion-correction of the PET images were performed using AIR (version 5.2.5). The 3D T1-weighted MPRAGE image was co-registered to PET images using SPM8. Delineation of region of interest was performed using PVE-lab [52], and performed on the individual’s T1-weighted MPRAGE image. The mean tissue time activity curves for the set of gray matter voxels within a region of interest (ROI) was extracted for kinetic modeling. Kinetic modeling using the simplified reference tissue model (SRTM) [53] with cerebellum (excluding vermis) was performed with PMOD software version 3.0 (PMOD, Zurich, Switzerland). The calculated non-displaceable binding potential (BP_ND_) served as the outcome measure for quantifying 5-HT_4_R binding. Mean BP_ND_ from bilateral caudate, putamen, amygdala, insula, vmPFC and dlPFC were extracted. The ROIs were chosen based on either an abundance of 5-HT_4_R binding (caudate and putamen) and/or their known relevance in emotion processing and the pathophysiology of MDD (i.e., amygdala, insula, vmPFC, dlPFC).

Lastly, the fMRI images were co-registered to PET space using SPM12. First, the 3D T1-weighted MPRAGE image in the individual’s MR space was co-registered to PET space and then applied to the functional MRI images. The contrast “all faces versus all shapes” from the functional MRI images was used to extract mean regional task-related brain reactivity estimates from the bilateral amygdala, insula, ventromedial prefrontal cortex and dorsolateral prefrontal cortex, delineated on the individual’s T1-weighted MPRAGE image using PVE-lab.

### Statistical analyses

#### Analyses of demographic, clinical, and radiotracer data

Analyses were conducted using R (version 4.1.2) [54]. Group differences in continuous (age, HAM-D_17_,MDI, BMI, weight-adjusted [^11^C]SB207145 injected mass (µg/kg)) and categorical (sex, 5-HTTLPR polymorphism) measures were assessed using Mann–Whitney *U*-tests and Fisher’s exact tests, respectively. All results were considered significant at *p* < 0.05.

#### Association between 5-HT_4_R binding and BOLD responses to emotional faces

We applied a linear latent variable model (LVM) using the *lava* package [55] implemented in R to first examine the association between 5-HT_4_R binding and BOLD responses to emotional faces. LVMs are flexible structural equation models, which allow us to model complex hierarchical structures and summarize multivariate data into a single latent variable, enabling ease of interpretation as well as reducing issues related to multiple testing of correlated variables. The PET variables were log transformed regional BP_ND_ values from the putamen, caudate, amygdala, insula, vmPFC and dlPFC; the fMRI variables were mean regional brain reactivity estimates from the amygdala, insula, vmPFC and dlPFC. Two latent variables, one representing the log-transformed 5-HT_4_R BP_ND_ estimates for the six PET ROIs (PET_LV_) and the other emotion-processing task-related mean regional brain reactivity estimates for the four fMRI ROIs (fMRI_LV_) were constructed. Age and sex were included as covariates on both PET_LV_ and fMRI_LV_; 5-HTTLPR and weight-adjusted [^11^C]SB207145 injected mass (µg/kg) were included as additional covariates on PET_LV_ due to their effect on 5-HT_4_R binding [56, 57]. The association between the two latent variables was modeled with a specific covariance parameter. Based on the estimated variance of the latent variables, this parameter was normalized to a Pearson correlation coefficient (*r*). We evaluated two models: the first was a common LVM for both MDD and healthy control group, irrespective of the group. In the second model, group specific LVMs were evaluated, which allowed the mean of each latent variable, and correlation between the latent variables to be group dependent. The difference in the correlation between the groups was assessed using a Wald test. Due to issues related to model convergence, covariate effects for the group specific LVMs were modeled directly on individual ROIs, rather than the latent variables, and the loading parameters were constrained to be equal for all individual ROIs relative to the same latent variable (except for the reference ROI where the loading is 1). Additional model paths were considered for the LVMs using the *modelsearch* function within *lava* and additional paths were added if the statistical significance of the Rao score test for the individual path was *p*_*FWER*_ < 0.05 (family wise error-corrected), adjusting for all possible paths. This procedure was repeated until no new model paths were supported.

## Results

### Demographic, clinical, genotype, and radiotracer information

Demographics, clinical and radiotracer information for patients with MDD and healthy controls are detailed in Table 1. There was no significant difference between patients with MDD and HC in age (*U* = 2193, *p* = 0.67), BMI (*U* = 2308, *p* = 0.63), 5-HTTLPR polymorphism (*p* = 0.84) or weight-adjusted [^11^C]SB207145 injected mass (*U* = 1973, *p* = 0.32). The cohort was predominately female (78.3%) and there were significantly more women in the healthy control group than in the patient group (*p* = 0.004). As expected, patients with MDD had statistically significantly higher scores on the MDI relative to the healthy controls (*U* = 4297, *p* < 0.001).

**Table 1:** Demographic, clinical, genotype and radiotracer information.

	Patients with MDD *N* = 88		Healthy controls *n* = 50		Significance
	Mean	SD	Mean	SD	*p*^a^
Age (years)	26.8	7.9	25.8	5.0	0.67
BMI (kg/m^2^)	24.6	5.6	23.5	3.5	0.63
MDI	34.2	7.4	17.0	15.2	<0.001
HAM-D_17_	22.8	3.4	—	—	
[11C]SB207145 injected mass (µg/kg)	0.013	0.014	0.015	0.016	0.32
	*N*	%	*N*	%	*p*^b^
Females	62	70.5	46	92	0.004
5-HTTLPR genotype (L_A_L_A_)	24	27.3	15	30	0.84

Demographic, clinical and radiotracer information for patients with major depressive disorder and healthy controls.

*MDD* Major Depressive Disorder, *BMI* Body Mass Index, *HAMD*_*17*_ 17-item Hamilton Depression Rating Scale, *MDI* Major Depressive Inventory, *p*^a^ *p*-value computed using Mann–Whitney U-test, *p*^b^ *p*-value computed using Fisher’s exact test.

### Association between 5-HT_4_R binding and BOLD responses to emotional faces

All six PET regions loaded strongly onto PET_LV_ (estimate range = 1.00:1.84, *p* < 1 × 10^−12^). Likewise, all four fMRI regions loaded strongly onto fMRI_LV_ (estimate range = 0.36:1.00, *p* < 1.41 × 10^−9^). Score tests supported an additional partial correlation between caudate and putamen (*p*_*FWER*_ = 1.60 × 10^−5^).

**Fig. 1:**
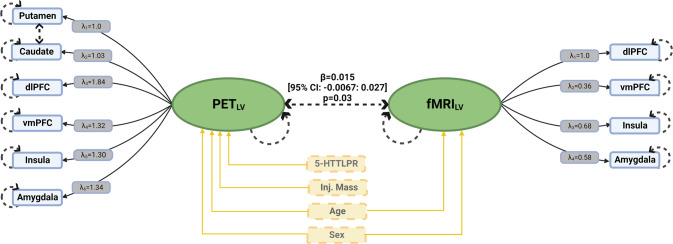
Latent variable model for the association between 5-HT_4_R binding and neural responses during processing of emotional stimuli. Figure shows the estimated latent variable model for the association between 5-HT_4_R binding and neural responses to emotionally salient stimuli. The green ellipses represent the latent variables PET_LV_ and fMRI_LV_. The dashed lines between caudate and putamen represent additional shared correlation. Circular dashed lines represent error estimates included in the model. β reflects the parameter estimate for the association between the two latent variables. _*λ*_ represents the loading of a given regional estimate onto its respective latent variable. Both PET_LV_ and fMRI_LV_ were adjusted for age and sex, and PET_LV_ was additionally adjusted for 5-HTTLPR polymorphism (5-HTTLPR) and weight-adjusted [^11^C]SB207145 injected mass (µg/kg) (Inj. Mass).

**Fig. 2:**
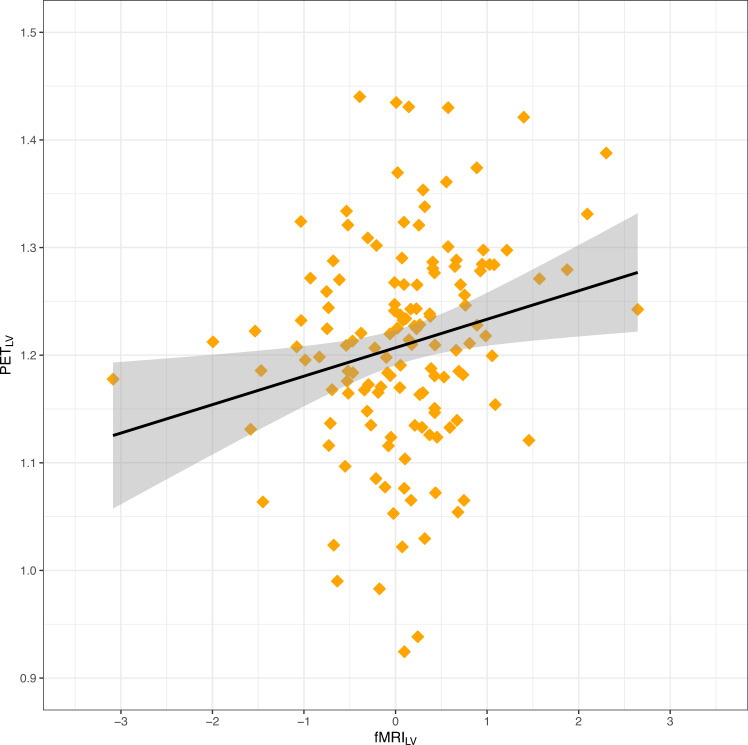
5-HT_4_R binding positively associated with neural response to emotional stimuli. Figure shows positive association between latent estimates of 5-HT_4_R binding (summarized as PET_LV_) and latent estimates of neural response to an emotional faces paradigm (summarized as fMRI_LV_) across all participants (*r* = 0.20, *p* = 0.03) obtained from the latent linear latent variable model. Both PET_LV_ and fMRI_LV_ were adjusted for age and sex, and PET_LV_ was additionally adjusted for 5-HTTLPR polymorphism and weight-adjusted [^11^C]SB207145 injected mass (µg/kg). The black line represents the slope and the gray shading represent 95% confidence intervals.

We observed a statistically significant positive association between 5-HT_4_R BP_ND_ (PET_LV_) and fMRI BOLD response to emotional faces (fMRI_LV_) across all participants (parameter estimate = 0.015, 95% CI = −0.0067: 0.027, *r* = 0.20, *p* = 0.03, Figs. 1 and 2). We did not observe any statistically significant effects of age, sex, genotype, or weight-adjusted [^11^C]SB207145 injected mass (µg/kg) on either of the LVs (estimate range = −0.95: 0.02, *p* > 0.07). In the group specific LVM evaluation, we did not observe that group significantly moderated the association between the two latent variables (parameter estimate difference = 0.002, 95% CI = −0.008: 0.013, *p* = 0.72). Additional observations from our model, as reported previously in related samples, included significantly lower 5-HT_4_R BP_ND_ (summarized by the PET_LV_) in MDD relative to HC [15], as well as no significant difference in BOLD response (summarized by the fMRI_LV_) between MDD and HC [48].

As mentioned in the Methods section, we made an assumption in the group specific model that the individual ROIs load on to the latent variables equally. This was due to lack of convergence in the model when we did not constrain the factor loadings. A sensitivity analysis showed that although the individual parameter estimates differed slightly, the group difference in the correlation between the latent variables was not statistically significant, similar to the results obtained when we constrained the factor loadings (*p* = 0.96 vs. *p* = 0.72).

## Discussion

In one of the largest single site PET/fMRI investigations to date, we evaluated the association between brain 5-HT_4_R binding and distributed brain responses to an emotional faces paradigm using a multimodal PET/fMRI brain imaging framework in 138 individuals, of whom 88 had MDD. We observed a statistically significant positive correlation between 5-HT_4_R binding and reactivity to emotional faces. However, there was no indication that this positive association differed between patients with MDD and healthy controls. These findings provide novel support for a link between serotonin neurotransmission and neural processing of emotionally salient stimuli. The lack of a group moderation effect suggests that, despite evidence for alterations in underlying serotonin neurotransmission in MDD, this coupling may be similar across groups.

This is the first in vivo examination of the relationship between 5-HT_4_R binding and brain responses to emotional faces in patients with MDD. Prior studies in healthy controls have demonstrated that serotonergic signaling, indexed either by 5-HT_1A_ or 5-HT_4_R binding, is linked to heightened responsivity to emotional faces in the amygdala. A study by Fisher and colleagues [30], for instance, found a negative association between 5-HT_1A_ auto-receptor binding and amygdala reactivity to emotional faces. Likewise, in a subsequent study, Fisher and colleagues [35] reported fluctuations in 5-HT_4_R binding over a 3-week period to be positively associated with changes in amygdala reactivity to emotional faces. While the amygdala is an important node in the emotion processing neural network, several lines of investigations have reliably shown co-activation of the prefrontal cortex and insula during processing of emotional cues, indicating that these regions also play a central role in emotion processing [20, 46, 58]. Our findings, therefore, build on the previous studies by Fisher and colleagues [30, 35] performed in healthy controls and show that the serotoninergic signaling may modulate responsivity not just in the amygdala, but also in a larger corticolimbic network subserving emotion processing across healthy controls and patients with MDD.

Several lines of investigations have posited that the coupling between serotonergic signaling and brain emotional reactivity may be altered in MDD, which may in-turn underlie risk for MDD [59]. Our current findings are *not* consistent with this model, rather, our data point to a preserved coupling between serotonin signaling and emotion processing in MDD. To our knowledge, only one other study, albeit using a small sample, has examined the association between serotoninergic signaling (via the 5-HT_1A_R) and fMRI responses to emotional stimuli directly in patients with MDD [59]. Post-hoc analyses conducted by Schenck et al. [59]. revealed that 5-HT_1A_R binding in the hippocampus was positively associated with hippocampal inhibitory connectivity in a sample of 27 patients with MDD, but not in their sample of 22 healthy controls. Whether the group difference in the coupling between serotonergic signaling and emotional reactivity is specific for 5-HT_1A_R or as a result of methodological differences needs further investigation.

We have previously shown in healthy humans that 5-HT_4_R binding assessed with [^11^C]SB207145 PET may be a putative inverse marker of central serotonin levels [51]. Preclinical evidence also supports an inverse association between 5-HT_4_R binding and serotonin levels [60]. Preclinical pharmacological studies have shown that administration of 5-HT_4_R agonists such as RS67333 and prucalopride increase dorsal raphe nucleus 5-HT neuron firing [61, 62]. Based on that interpretation, our findings suggest that low brain serotonin levels are associated with heightened corticolimbic reactivity to emotional stimuli in humans.

It may also be that our observed association reflects a more direct relation between 5-HT_4_R signaling and corticolimbic reactivity to emotional stimuli. Such an effect supports the notion that new pharmacological antidepressant treatments that work on the 5-HT_4_R could also help in modulating corticolimbic circuitry subserving aberrant emotional response, a strong clinical risk factor of recurrence, relapse, and suicide risk in individuals affected by MDD [36–38]. Our finding also suggests that psychotherapeutic interventions such as cognitive behavioral therapy shown to modulate brain circuitry underlying emotion processing and regulation [63] could help increase brain serotonin level in patients with MDD. A recent study showed that short term (6 days) treatment with low-dose prucalopride (5-HT_4_R agonist) was associated with improved accuracy in gender recognition as well as alterations in cortical brain responses during an fMRI emotion processing task [64]. Future clinical trials with pharmacological agents that target the 5-HT_4_R system, carefully considering treatment dose and duration, are needed to confirm whether directly targeting 5-HT_4_R can improve emotion processing biases in MDD.

These findings should be considered in the context of its limitations. First, our overall sample had an over-representation of women, and our healthy control group had proportionally more women than in the MDD group. We are unaware of prior studies indicating that sex moderated the association between serotonergic signaling and brain function, and we further included sex as a covariate in all analyses. Notwithstanding, the sex distribution in our study aligns with the over-representation of females among patients with MDD. Second, we did not observe group differences in brain responses to the emotional faces fMRI paradigm, as reported in this cohort previously [48]. This may have influenced the ability to detect a significant interaction effect. Third, it is not clear the extent to which [^11^C]SB207145 binding reflects central brain serotonin levels, 5-HT_4_R signaling, or a combination. Regardless, its relation to the brain response to emotional stimuli reinforces the relevance of serotonin neurotransmission in emotion processing and the need to delineate relevant receptor mechanisms that can be targeted with novel drugs. Lastly, since we did not include individuals older than 60 years, our results may not be generalizable to older populations.

In conclusion, in the largest single site in vivo PET/fMRI study of associations between serotonergic signaling and brain function conducted to date, we show for the first time that high 5-HT_4_R binding is associated with heightened responses to emotional stimuli in corticolimbic regions in individuals, and this association seems to be preserved in MDD.
